# Correlation Between Body Mass Index and Periodontitis: A Clinical and Biochemical Analysis

**DOI:** 10.7759/cureus.62279

**Published:** 2024-06-12

**Authors:** Johnisha Harris, Arvina Rajasekar

**Affiliations:** 1 Periodontics, Saveetha Dental College and Hospitals, Saveetha Institute of Medical and Technical Sciences, Saveetha University, Chennai, IND

**Keywords:** visfatin, periodontitis, periodontal diseases, obesity, adipokine

## Abstract

Introduction

Obesity is the excessive deposition of body fat in relation to lean body mass. In this research, its relation to periodontitis has been analysed using clinical and biochemical parameters. The current study assessed the correlation between body mass index (BMI) and periodontitis using salivary visfatin levels.

Materials and methods

Sixty participants (33 males and 27 females) were categorised into three different groups according to BMI: group 1: normal weight (n=20); group 2: overweight (n=20); and group 3: obese (n=20). Clinical parameters such as probing pocket depth (PPD) and clinical attachment level (CAL) were recorded. Salivary samples were collected and assessed for salivary visfatin levels with the aid of a human visfatin enzyme-linked immunosorbent assay (ELISA) kit. The results were assessed using IBM SPSS Statistics for Windows, Version 23.0 (Released 2015; IBM Corp., Armonk, New York, United States).

Results

The PPD, CAL, and salivary visfatin levels were higher in group 3, followed by groups 2 and 1, and were statistically significant (p=0.000). The correlation between visfatin and PPD (r=0.962) and visfatin and CAL (r=0.978) was strongly positive and statistically significant.

Conclusion

This study demonstrates a strong positive correlation between BMI and periodontitis. Moreover, salivary visfatin can be considered a diagnostic marker for periodontal diseases.

## Introduction

Periodontitis is a chronic inflammatory disease affecting the supporting tissues of teeth. This inflammation is mainly caused by bacteria and their by-products that accumulate in the oral cavity, leading to an immune response that eventually damages the periodontal tissues. This prolonged inflammatory response leads to the release of pro-inflammatory cytokines, which contribute to the breakdown of connective tissues and bone loss [1]. Several risk factors that increase the susceptibility to periodontitis are smoking, genetics, systemic conditions like diabetes, and obesity [2,3]. Among these, the relationship between obesity and periodontitis has been studied for the past few years. Obesity refers to the excessive deposition of body fat in addition to lean body mass, often resulting from a combination of genetic, environmental, and lifestyle factors [4]. It is calculated in terms of body mass index (BMI) by measuring the person's height and weight. Obesity is linked with health hazards like cardiovascular disease, diabetes, musculoskeletal disorders, etc. Periodontitis can also possibly be added to this list of risk factors. The potential relationship between obesity and periodontitis arises from similar risk factors and systemic inflammation [5].

Obesity is also characterised by chronic, low-grade inflammation throughout the body. Adipose tissue that produces various inflammatory molecules, including cytokines and adipokines, can affect the immune response and exacerbate inflammation. This systemic inflammation may contribute to the progression of periodontitis and worsen its severity [6]. One such adipokine that has gained attention for playing a major and equal role in periodontitis and obesity is visfatin [7]. Visfatin, also known as nicotinamide phosphoribosyltransferase (NAMPT), is a protein that helps support the growth and development of immune cells. However, visfatin also exhibits additional functions and has garnered attention for its potential involvement in various physiological and pathological processes [8]. It acts as a cytokine, promoting the production of pro-inflammatory molecules and influencing immune cell function. It has been found to be elevated in certain inflammatory conditions, like rheumatoid arthritis and periodontitis [9]. Hence, this study was done to assess the correlation between BMI and periodontitis using salivary visfatin levels.

## Materials and methods

This observational study was done by recruiting 60 outpatients who reported to the Department of Periodontics, Saveetha Dental College and Hospitals, Chennai, India, after approval by the Institutional Human Ethical Committee (IHEC/SDC/PERIO-2103/23/072). It was conducted after explaining the protocol and getting informed written consent from the subjects. The sample size was calculated to be 20 participants per group using the mean and standard deviation values from the previous study [10]. A total of 60 participants, including 33 males and 27 females, were split into three groups, with 20 participants in each group. The average ages of the subjects in all groups were between 21 and 35 years old. The patients were categorised with respect to their BMI as group 1: normal weight (18.5-24.9), group 2: overweight (25-29.9), and group 3: obese (>30) based on the criteria given by the National Institutes of Health. Patients aged between 25 and 60 years with no systemic diseases and at least 20 remaining teeth in the oral cavity, excluding the third molars, were included. Patients with systemic diseases, smokers, alcoholics, pregnant and lactating women, and those with a history of periodontal therapy within the past six months were excluded from the study.

Clinical examination

**Figure 1:**
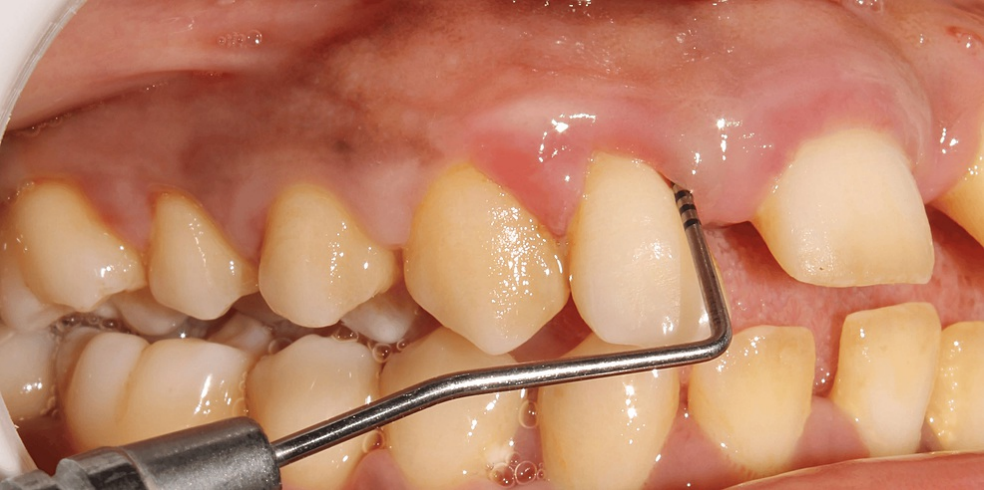
Assessment of clinical parameters

On clinical examination, probing pocket depth (PPD) and clinical attachment level (CAL) were assessed using the UNC-15 periodontal probe (Figure 1). The mean value was taken into consideration. The BMI was calculated based on each subject's weight in kilograms divided by the square of his or her height in meters (kg/m^2^).

Sample collection 

**Figure 2:**
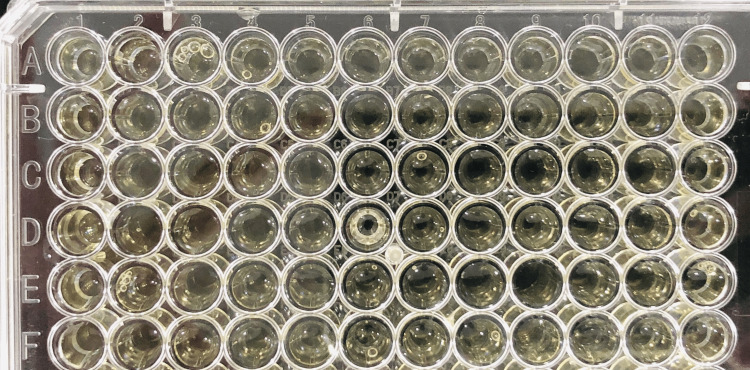
Transferring samples to ELISA kit ELISA: enzyme-linked immunosorbent assay

The subjects were instructed to fast for at least three hours before collecting the saliva sample. From each participant, 5 ml of unstimulated saliva was collected by the spitting method. After collecting the saliva in sterile containers, they were stored at -80°C until the experiment started. The frozen saliva samples were used for biochemical analysis within six months. Each 5 ml saliva sample was transferred to a sterile container, and the levels of visfatin were assessed with the aid of the Elabscience human visfatin enzyme-linked immunosorbent assay (ELISA) kit (Figure 2). This kit works on the principle of sandwich ELISA. 100 μL of samples were added to the wells and incubated for 90 minutes at 37°C. The liquid was discarded immediately, and 100 μL biotinylated detection Ab working solution was added to each well. They were later incubated for 60 minutes at 37°C. The plates were washed thoroughly three times. To this mixture, 100 μL HRP conjugate working solution was added, incubated for 30 minutes at 37°C, and washed five times. Finally, add 90 μL substrate reagent and incubate for 15 minutes at 37°C. Eventually, after adding 50 μL stop solution, the plate was read at 450 nm immediately. The results were measured in terms of ng/mL. A minimum of 0.19 ng/mL of visfatin should be present to be detected by the kit.

Statistical analysis 

Statistical analysis was performed using IBM SPSS Statistics for Windows, Version 23.0 (Released 2015; IBM Corp., Armonk, New York, United States). Clinical variables and salivary levels of visfatin were compared among three groups using one-way ANOVA and pairwise comparison by Tukey's Honestly Significant Difference (HSD) post hoc analysis. The correlation was performed using Pearson's correlation. A p-value of less than 0.05 was considered statistically significant.

## Results

In this study, 20 normal-weight individuals in group 1 (27.3±4.73 years), 20 overweight individuals in group 2 (26.4±5.2 years), and 20 obese individuals in group 3 (28.3±4.8 years) were included (Table 1).

**Table 1:** Demographic details

Parameter	Group 1 (n=20)	Group 2 (n=20)	Group 3 (n=20)
Age (years)(mean±SD)	27.3±4.73	26.4±5.2	28.3±4.8
Sex (male/female) (n)	11/9	11/9	11/9

Table 2 depicts the comparison of PPD, CAL, and salivary visfatin levels between the three groups using ANOVA. The PPD, CAL, and salivary visfatin levels were higher in group 3, followed by groups 2 and 1, and were statistically significant (p=0.000).

**Table 2:** Intergroup comparison of PPD, CAL, and salivary visfatin levels between the three groups *The mean difference is significant at the 0.05 level PPD: probing pocket depth; CAL: clinical attachment level

Variables	Group 1 (mean±SD)	Group 2 (mean±SD)	Group 3 (mean±SD)	P-value
PPD (mm)	3.31±1.68	4.67±1.39	6.6±2.02	.000*
CAL (mm)	1.52±2.79	4.68±2.25	6.49±3.04	.000*
Salivary visfatin (ng/ml)	24.35±6.07	29.65±4.47	33.4±5.93	.000*

In pairwise comparison, in terms of CAL and visfatin levels, a statistically significant difference was observed only between groups 1 and 3 and groups 1 and 2 (p=0.000), which is depicted in Table 3.

**Table 3:** Pairwise comparison of PPD, CAL, and visfatin between the three groups *The mean difference is significant at the 0.05 level PPD: probing pocket depth; CAL: clinical attachment level

Groups		PPD		CAL		Visfatin	
		Mean difference	P-value	Mean difference	P-value	Mean difference	P-value
Group 1	Group 2	-1.360	.040^*^	-3.160	.002^*^	-5.300	.010^*^
	Group 3	-3.295	.000^*^	-4.970	.000^*^	-9.100	.000^*^
Group 2	Group 1	1.360	.040^*^	3.160	.002^*^	5.300	.010^*^
	Group 3	-1.935	.002^*^	-1.810	.098	-3.800	.085
Group 3	Group 1	3.295	.000^*^	4.970	.000^*^	9.100	.000^*^
	Group 2	1.935	.002^*^	1.810	.098	3.800	.085

Table 4 depicts Pearson's correlation of salivary visfatin levels with clinical parameters. Visfatin and PPD (r=0.962) and visfatin and CAL (r=0.978) revealed a strong positive correlation with a statistically significant difference.

**Table 4:** Correlation of visfatin with PPD and CAL **Correlation is significant at the 0.01 level (two-tailed) PPD: probing pocket depth; CAL: clinical attachment level

Correlation variable	Correlation coefficient (r)	P-value
PPD vs visfatin	0.962^**^	.000
CAL vs visfatin	0.978^**^	.000

## Discussion

This study correlated the BMI of normal, overweight, and obese people with periodontitis. Periodontal health has a deep impact on day-to-day life, from having a confident smile to having low self-esteem. As periodontitis progresses, it can cause changes in the appearance of the teeth. Gingival recession and tooth loss can lead to an unattractive appearance [11]. These changes can have a significant impact on a person's self-esteem and overall confidence. It's important to note that periodontitis is preventable and treatable if it can be treated at an earlier stage. But clinical and radiographic examinations only help in diagnosing the current stage of the disease [12]. To help in predicting the disease, biomarkers play a major role. Especially in periodontitis, saliva and gingival crevicular fluid serve as good sources of biomarkers [13]. Saliva collection is easy, non-invasive, and minimally time-consuming [14,15].

The results of the study by Kumar et al. [16] demonstrated similar results, showing high visfatin levels in chronic periodontitis patients. Abolfazli et al. [17] evaluated serum as well as salivary visfatin levels in patients with chronic periodontitis before and after non-surgical periodontal therapy (NSPT) and showed a significant decrease in visfatin levels post-NSPT. Salivary visfatin level changes were more significant after periodontal therapy. This showed the association between periodontal inflammation and the progression of the disease with salivary and serum visfatin levels. Özcan et al. [18] studied the association between salivary visfatin levels and periodontitis. Increased levels of salivary visfatin were noted among gingivitis and periodontitis patients compared to healthy subjects [18].

Furthermore, immunohistochemical analysis in chronic and aggressive periodontitis patients using gingival tissue samples done by Tabari et al. [19] revealed that the degree of inflammation and expression of visfatin were significantly higher in periodontitis patients than periodontally healthy individuals. Also, the association between inflammation and visfatin was strongly positive and statistically significant in aggressive periodontitis. Yadalam et al. [20] suggested the possible role of salivary visfatin in the pathogenesis of periodontal diseases. Also, Saseendran et al. [21] demonstrated that salivary visfatin levels were higher among chronic periodontitis patients than periodontally healthy subjects. This demonstrates the fact that levels of salivary visfatin can be used as an inflammatory biomarker in the diagnosis of periodontal diseases. Similar findings were observed in various studies [22-24]. The findings of the present study are in alignment with previous studies showing a positive relationship between periodontal clinical parameters and salivary visfatin levels.

Limitations

A limited study population and the cross-sectional study design are two of the disadvantages of the study. Consequently, more extensive research is required to establish the relationship between obesity and periodontal disease as well as to comprehend the function of visfatin in the pathogenesis of periodontitis.

## Conclusions

The results of the current study show a strong positive correlation between BMI and periodontitis in terms of periodontal parameters and salivary visfatin levels. This proves the association between periodontitis and obesity. Also, this study validates the potential use of salivary visfatin as a marker for the diagnosis and treatment of periodontal diseases. However, the molecular and cellular pathways of visfatin in the etiopathogenesis of periodontitis should be explored in future studies.

## References

[1] Listgarten MA (1986). Pathogenesis of periodontitis. J Clin Periodontol.

[2] Kandhan TS, Rajasekar A (2020). Prevalence of periodontal diseases among patients with and without systemic diseases - a retrospective study. J Complement Med Res.

[3] Anjum S, Rajasekar A (2022). Assessment of independent variables of periodontal disease among selected South Indian population. J Adv Pharm Technol Res.

[4] Kwon T, Lamster IB, Levin L (2021). Current concepts in the management of periodontitis. Int Dent J.

[5] Ganesan SM, Vazana S, Stuhr S (2021). Waistline to the gumline: relationship between obesity and periodontal disease-biological and management considerations. Periodontol 2000.

[6] Kim CM, Lee S, Hwang W, Son E, Kim TW, Kim K, Kim YH (2022). Obesity and periodontitis: a systematic review and updated meta-analysis. Front Endocrinol (Lausanne).

[7] Dakroub A, Nasser SA, Kobeissy F (2021). Visfatin: an emerging adipocytokine bridging the gap in the evolution of cardiovascular diseases. J Cell Physiol.

[8] Mopidevi A, Penmetsa GS, Dwarkanath CD, Dubba K, Gadde P (2019). Salivary visfatin concentrations in patients with chronic periodontitis: an analysis before and after periodontal therapy. Indian J Dent Res.

[9] Jia R, Zhang Y, Wang Z, Hu B, Wang Z, Qiao H (2023). Association between lipid metabolism and periodontitis in obese patients: a cross-sectional study. BMC Endocr Disord.

[10] Rajasekar A (2023). Correlation of salivary visfatin levels in obese and non-obese population with periodontal status. J Oral Biol Craniofac Res.

[11] Bayani M, Pourali M, Keivan M (2017). Possible interaction between visfatin, periodontal infection, and other systemic diseases: a brief review of literature. Eur J Dent.

[12] Wong LB, Yap AU, Allen PF (2021). Periodontal disease and quality of life: umbrella review of systematic reviews. J Periodontal Res.

[13] Goh V, Hassan FW, Baharin B, Rosli TI (2022). Impact of psychological states on periodontitis severity and oral health-related quality of life. J Oral Sci.

[14] Arias-Bujanda N, Regueira-Iglesias A, Balsa-Castro C, Nibali L, Donos N, Tomás I (2020). Accuracy of single molecular biomarkers in saliva for the diagnosis of periodontitis: a systematic review and meta-analysis. J Clin Periodontol.

[15] Verhulst MJ, Teeuw WJ, Bizzarro S (2019). A rapid, non-invasive tool for periodontitis screening in a medical care setting. BMC Oral Health.

[16] Kumar V, Pratap M, Sharma S, Singh K, Saimbi CS (2019). Evaluation of salivary levels of visfatin in obese patients with chronic periodontitis. Indian J Dent.

[17] Abolfazli N, Jabali S, Saleh Saber F, Babaloo Z, Shirmohammadi A (2015). Effect of non-surgical periodontal therapy on serum and salivary concentrations of visfatin in patients with chronic periodontitis. J Dent Res Dent Clin Dent Prospects.

[18] Özcan E, Saygun NI, Serdar MA, Kurt N (2015). Evaluation of the salivary levels of visfatin, chemerin, and progranulin in periodontal inflammation. Clin Oral Investig.

[19] Tabari ZA, Keshani F, Sharbatdaran M, Banishahabadi A, Nejatifard M, Ghorbani H (2018). Visfatin expression in gingival tissues of chronic periodontitis and aggressive periodontitis patients: an immunohistochemical analysis. Dent Res J (Isfahan).

[20] Yadalam PK, Kalaivani V, Fageeh HI (2022). Future drug targets in periodontal personalised medicine-a narrative review. J Pers Med.

[21] Saseendran G, Abraham S, Nair AM, Reejamol MK (2021). Comparative evaluation of salivary visfatin levels in healthy and periodontally diseased patients before and after scaling and root planing. J Pharm Bioallied Sci.

[22] Paul R, Suresh S, Sudhakar U, Jean C, Fernandez KJ (2020). Evaluation of association between Porphyromonas gingivalis and visfatin levels in chronic periodontitis patients. J Indian Soc Periodontol.

[23] Rajasekar A, Ganapathy D (2023). Effectiveness of nonsurgical periodontal therapy on salivary visfatin: a clinical and biochemical analysis. World J Dent.

[24] Pradeep AR, Raghavendra NM, Sharma A (2012). Association of serum and crevicular visfatin levels in periodontal health and disease with type 2 diabetes mellitus. J Periodontol.

